# Life Cycle Assessment support to environmental ambitions of EU policies and the Sustainable Development Goals

**DOI:** 10.1002/ieam.4586

**Published:** 2022-02-21

**Authors:** Esther Sanyé‐Mengual, Serenella Sala

**Affiliations:** ^1^ European Commission, Joint Research Centre (JRC) Ispra Italy

**Keywords:** Environmental decoupling, European Green Deal, Life Cycle Assessment, Planetary Boundaries, Sustainable Development Goals

## Abstract

The European Green Deal and the Sustainable Development Goals (SDGs) ask for a more holistic approach to production and consumption along value chains. The role of Life Cycle Assessment (LCA) in supporting policy design and monitoring is then pivotal to achieving policy ambitions. This paper explores the potential support of LCA to EU (European Union) policies and the SDGs, considering also the Planetary Boundaries (PBs) framework. The assessment focuses on (a) the relationship between LCA, the SDGs, and the European Green Deal; (b) the potential use of LCA in support of the monitoring of SDG12 and the environmental impacts of production and consumption; and (c) the relevance of an absolute sustainability dimension, including the integration of the PBs framework in EU policy and the SDGs. Results highlight that the interlinkages between LCA, EU policy, SDGs, and the PBs can be classified as existing, missing, or existing depending on the LCA framework. In general, LCA was identified to strengthen and further enable EU policies toward achieving the SDGs while remaining within the physical limits of the planet. This is because LCA can be a pivotal method to quantify and assess environmental impacts of value chains and consumption patterns, enabling the evaluation of their implication on environment‐related SDGs and assessing them against the PBs. The example of the Consumption Footprint highlights that the concept and Life Cycle Impact Assessment method of an LCA framework can determine the linkage among EU policy, SDGs, and the PBs. *Integr Environ Assess Manag* 2022;18:1221–1232. © 2022 The Authors. *Integrated Environmental Assessment and Management* published by Wiley Periodicals LLC on behalf of Society of Environmental Toxicology & Chemistry (SETAC).

## Key Points

- Life cycle assessment might have a crucial role in addressing the Sustainable Development Goals (SDGs) in an integrated manner, linking the modeling of production and consumption with the environmental related goals of the SDGs.
- Qualitative interlinkages among SDGs could be quantified by implementing a life cycle assessment based framework.
- Life cycle based indicators resulting from combining product characteristics with consumption intensities are pivotal to monitor evolution of impacts over time.

## INTRODUCTION

Current production and consumption patterns have been identified as the source of environmental impacts hampering global environmental quality (United Nations Environment, 2019). Due to the large impacts caused by human interventions, we are now living in the Anthropocene (Crutzen, 2002; Steffen et al., 2007), a new geological era where human‐driven irreversible changes are happening. The current COVID‐19 pandemic has emphasized the close interdependency between society, production, and consumption patterns and the state of the environment, while the consequences of the pandemic on the environment are still uncertain (EEA, 2020): Lockdowns around the globe may have positive temporary effects (e.g., decreased carbon footprint; Rugani & Caro, 2020), but there is a higher generation of single‐use plastic and medical waste (Klemeš et al., 2020).

At the international level, the United Nations (UN) adopted the 2030 Agenda for Sustainable Development and the 17 Sustainable Development Goals (SDGs) (UN, 2015) with the aim of enabling a sustainable pathway where environmental impacts are minimized while welfare is maximized. The SDGs address global challenges, including specific targets and a set of indicators, ranging from human health and equity to terrestrial and marine ecosystems.

At the EU level, policy development is aligned with the SDGs framework and sustainable development is monitored with a set of SDG indicators based mainly on available EU statistics (Eurostat, 2020a). In line with this, the European Commission (EC) published political guidelines highlighting the need for a transition toward a healthier and more sustainable Europe (von der Leyen, 2019), including “becoming the world's first climate‐neutral continent,” changing the way EU's “produce, consume and trade,” “sustainable food along the whole value chain,” having “a zero‐pollution ambition,” and proposing a New Circular Economy Action Plan focusing on sustainable resource use.

For this purpose, the European Green Deal was published as the new strategy of the EC aimed at transforming the EU with a modern, resource‐efficient, and competitive economy (EC, 2019a), a transformation to set the path toward achieving SDGs while ensuring a decoupling between economic growth and resource use. The European Green Deal highlighted the relevance of a holistic approach to the value chain and consumption‐based approach. Ideally, trade and the embedded environmental and social impacts in imported goods and services should be accounted for increasingly more, allowing for the identification of potential spill‐over effects in other world regions due to EU consumption. For example, the need to address entire value chains was at the basis of the Farm to Fork Strategy regarding food systems (EC, 2020a) and specific spill‐over effects were addressed in the communication on deforestation (EC, 2019b). Furthermore, the European Green Deal also mentions the need to pay attention to potential trade‐offs between different sustainability aspects.

In this context, Life Cycle Thinking (LCT) and Life Cycle Assessment (LCA) (ISO, 2006a, 2006b, 2017, 2020a, 2020b) may play a significant role in assessing value chains in support of EU policies and toward SDGs. Life Cycle Assessment is a comprehensive quantitative method to assess the environmental impacts due to resource use and emissions to the environment along the life cycle of systems, products, and processes, thereby considering the entire value chain: from extraction of raw materials to end‐of‐life management. This approach enables identification of potential trade‐offs between life cycle stages (Sala, Benini, et al., 2019), for example, improving energy efficiency in the use phase while increasing raw material extraction, and among environmental impact categories, for example, reducing climate impacts while increasing toxicity risks.

Life Cycle Assessment is one of the approaches included in the Better Regulation Toolbox, which compiles the best tools to use along the policymaking cycle toward better regulation (EC, 2015, 2021a). The systemic approach of LCA enables this method to contribute to the different stages of the policy cycle (i.e., from policy anticipation and problem definition to policy evaluation), leading to the integration of LCA into several EU policies (Sala et al., 2016, 2021). With the increasing role of SDGs in EU policy and the prominence of environmental ambitions in the European Green Deal, analyzing the relevance of LCA in EU policies addressing SDGs is key.

The potential use of LCA for monitoring the SDGs was recently explored in the literature. The impact assessment models underpinning environmental LCA and social LCA (S‐LCA) could address a significant number of SDGs (Sala, 2019). For example, from a supply‐chain perspective, S‐LCA can potentially be used to assess the social impacts of raw materials (Mancini et al., 2019). The UN Life Cycle Initiative project “Linking the UN Sustainable Development Goals to life cycle impact pathway frameworks” is addressing the implementation of LCA to monitor SDGs at the corporate level (Weidema et al., 2020).

Finally, the need to remain within the limits of the planet and the relevance of considering the physical boundaries of ecosystems have been mentioned since the seventh Environmental Action Plan (European Parliament and Council, 2013) and reiterated in the European Green Deal. Therefore, absolute sustainability stands out as a required approach to address sustainable development, contrary to relative assessments that can disguise increases of environmental impacts in absolute terms (Hauschild, 2015). In this context, the Planetary Boundaries (PBs) framework (Rockström et al., 2009; Steffen et al., 2015) is a well‐known concept where the “safe operating space” for human development is defined and can provide a quantitative reference to be considered as a threshold in an absolute sustainability perspective, where physical limits are known.

This paper aims to assess the potential role of LCA in EU policies addressing SDGs by exploring the interlinkages between LCA, EU policy, and SDGs in the context of the environmental dimension of sustainability and the PBs framework. In particular, the assessment focused on exploring whether LCA‐based frameworks and indicators can strengthen the linkage between EU policies, the SDGs, and the PBs, thereby enabling policymaking to reach international sustainability ambitions considering the physical limits of the planet. Three main aspects were examined in detail: (a) the relationship between LCA, the SDGs, and the European Green Deal; (b) the potential use of LCA in support of the monitoring of SDG12 and the environmental impacts of production and consumption; and (c) the relevance of an absolute sustainability dimension, including the integration of the PBs framework in EU policy and the SDGs. For this purpose, the Consumption Footprint is explored as an example of an LCA‐based framework designed in the SDGs context.

## MATERIALS AND METHODS

### Assessing links between SDGs, EU policies, LCA, and the PBs

**Figure 1:**
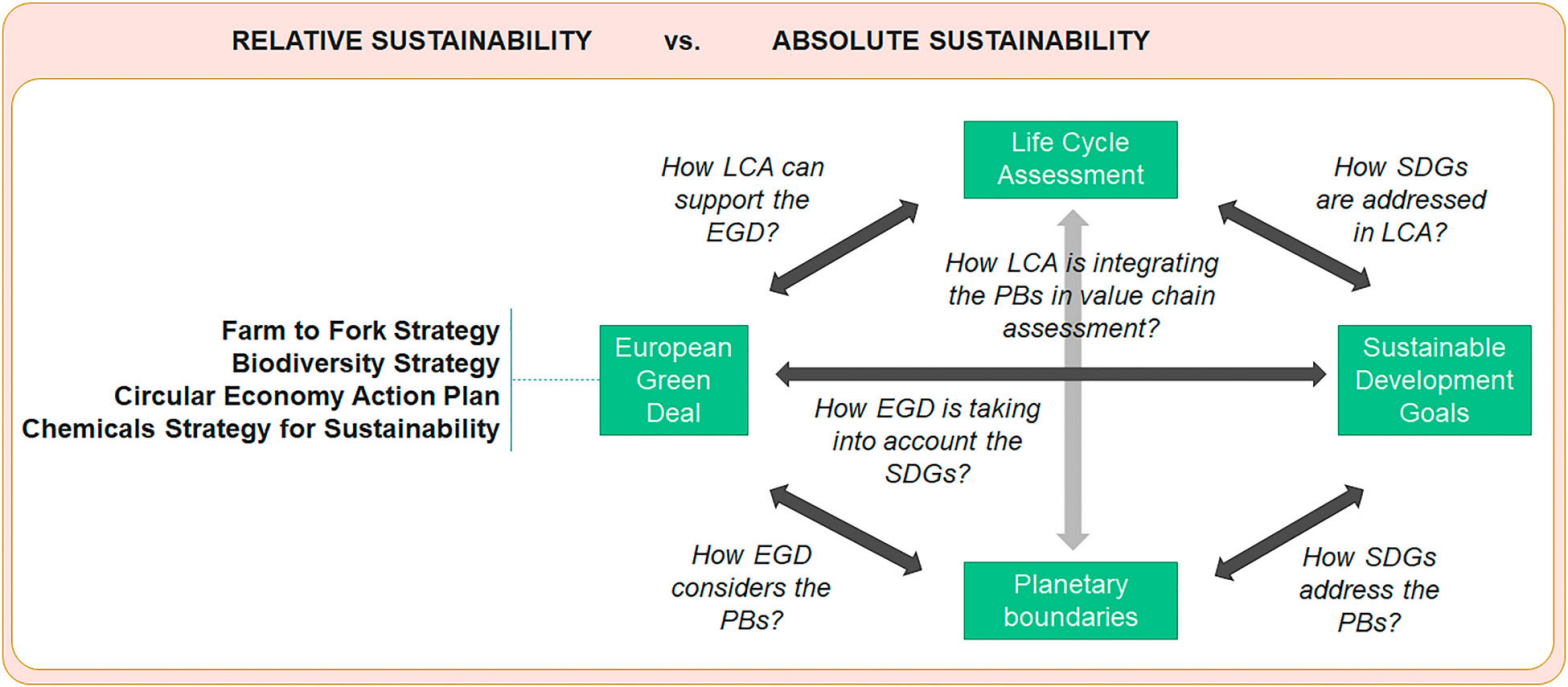
Analysis framework for assessing the support of Life Cycle Assessment (LCA) to the European Union's policies addressing Sustainable Development Goals (SDGs) and their relationship with the Planetary Boundaries (PBs) framework, considering both relative and absolute sustainability

The assessment of the potential support of LCA to EU policies toward achieving the SDGs relies on the analysis of the interrelationships between these three elements and their links to the PBs framework (Figure 1). The assessment covered mainly environment‐related SDGs due to the scope of LCA, which is addressing environmental impacts. Regarding the EGD, the study analyzed specific policies, namely, the Farm to Fork Strategy (EC, 2020a), the Biodiversity Strategy (EC, 2020b), the Circular Economy Action Plan (EC, 2020c), and the Chemicals Strategy for Sustainability (EC, 2020d). These policies were selected due to (a) the level of maturity (i.e., published communications), (b) previous identification in review studies of LCA use in policy, and (c) transversality among environmental issues, thereby associated with four different ambitions of the European Green Deal, namely, Preserving Europe's natural capital, Transition to a circular economy, A zero pollution Europe, and Farm to Fork.

The assessment pays particular attention to sustainable consumption and production by evaluating the role of the LCA‐based Consumption Footprint indicator (Sala, Benini, et al., 2019; Sala & Castellani, 2019) in supporting SDG12 (on ensuring sustainable production and consumption patterns). Two different levels of sustainability are addressed mirroring the concept of weak and strong sustainability (Neumayer, 2003): (a) relative sustainability, which addresses progress with respect to a previous situation (e.g., time trends, decoupling assessment) or between two systems and (b) absolute sustainability, which considers thresholds that should not be trespassed (e.g., PBs framework).

### The Consumption Footprint indicator

The Consumption Footprint is an LCA‐based indicator to assess the environmental impacts of EU household consumption from a bottom‐up perspective, namely, using process‐based LCA of representative products of consumption (Sala, Benini, et al., 2019; Sala & Castellani, 2019; Sala & Sanyé Mengual, 2022). The indicator encompasses five key areas of consumption in the EU: food (Castellani et al., 2017b; Notarnicola et al., 2017), mobility (Castellani et al., 2017a), housing (Baldassarri et al., 2017; Lavagna et al., 2018), household appliances (Hischier et al., 2020; Reale et al., 2019), and household goods (Castellani et al., 2019, 2021). Progressively refined and extended in scope over time, the Consumption Footprint currently includes around 165 representative products, for which the environmental burdens are quantified based on the consumption intensity in a given year (i.e., apparent consumption) according to production and trade statistics (e.g., Eurostat, 2020b, 2020c) and a life cycle inventory (LCI) model of the environmental pressures associated with their supply chain (i.e. resource use, emissions to the environment) (Equation 1). The most updated data are available at the Consumption Footprint Platform (EC‐JRC, 2021b).

(1) $$\;\mathrm{Consumption}\;\mathrm{Footprint}=\sum_{i=0}^{n}{\mathrm{Consumption}\;\mathrm{intensity}}_{i}\times {\mathrm{Environmental}\;\mathrm{impact}}_{i}=\sum_{i=0}^{n}{\left(\mathrm{Production}+\mathrm{Import}-\mathrm{Exports}\right)}_{i}\times {\mathrm{Environmental}\;\mathrm{impact}}_{i}.$$

Note that earlier versions of the Consumption Footprint indicator were termed as Consumer Footprint. In the current framework, the indicator is termed as Consumption Footprint and is used for country‐level and macroscale assessments based on consumption statistics (Sala & Sanyé Mengual, 2022). The bottom‐up approach of the original Consumer Footprint was retained as the reference method for consumption at a macroscale (i.e., it has the ambition to address impact at EU and country scale). Therefore, the term is now used in coherence with this methodological choice. The term Consumer Footprint is limited to the Consumer Footprint Calculator (EC‐JRC, 2021a), where individual citizens use their own consumption data to assess the environmental impacts of their lifestyle. From an SDGs perspective, both Consumption and Consumer Footprint approaches have the same coverage of SDGs.

The Environmental Footprint (EF) (EC, 2021b) is implemented in the life cycle impact assessment (LCIA) step for calculating the environmental impacts associated with the environmental pressures based on specific impact assessment models. The EF method includes 16 midpoint impact categories: climate change (CC); ozone depletion (ODP); human toxicity, noncancer (HTOX_nc); human toxicity, cancer (HTOX_c); particulate matter (PM); ionizing radiation, human health (IR); photochemical ozone formation, human health (POF); acidification (AC); eutrophication, terrestrial (TEU); eutrophication, freshwater (FEU); eutrophication, marine (MEU); ecotoxicity, freshwater (ECOTOX); land use (LU); water use (WU); resource use, fossils (FRD); and resource use, minerals, and metals (MRD).

### The PBs

The PBs framework (Rockström et al., 2009; Steffen et al., 2015) is a well‐known concept where the “safe operating space” for human development is defined based on the planet's biophysical processes, thereby providing a science‐based reference from which human interventions can result in ecosystems reaching tipping points. Therefore, this framework provides a quantitative reference to be considered as the threshold in an absolute sustainability perspective. The PBs framework considers nine among the Earth system processes and has been adapted to the 16 environmental impact categories of the EF method through the development of LCIA‐based PBs (Sala et al., 2020).

## RESULTS AND DISCUSSION

This section presents and discusses the main results of the analysis, as follows: (i) the relationship between LCA, the European Green Deal, and the SDGs; (ii) the potential use of LCA for monitoring SDG12 and the environmental impacts of production and consumption; and (iii) the assessment of SDGs from an LCA approach considering both relative and absolute sustainability perspectives, also paying attention to the integration of the PBs framework into EU policies and the SDGs.

### Interrelationships between LCA, EU policies, and SDGs

Focusing on some representative EU policies, the assessment of the Farm to Fork Strategy, the Biodiversity Strategy, the Circular Economy Action Plan, and the Chemicals Strategy for Sustainability highlight the broad coverage of environment‐related SDGs and the relevance of an LCA perspective (Table 1). Further details on the link between EU policy and their link to SDGs can be found in the KnowSDGs platform (EC‐JRC, 2020). Although the assessed policies also address social and economic aspects, this study focused on human health and ecosystem quality aspects associated with the environmental impacts within the scope of LCA, leading to a total of 10 covered SDGs.

**Table 1:** Coverage of environment‐related Sustainable Development Goals (SDGs) and relevance of Life Cycle Assessment (LCA) in the European Union's (EU) policies

EU policy	SDG coverage	Relevance of LCA
Farm to Fork Strategy	SDG2: sustainable food production SDG3: healthy diets SDG6: freshwater ecosystems SDG12: reduced food waste SDG14: marine ecosystems SDG15: land ecosystems SDG13: climate action	Reduced environmental and climate footprint of the EU food system, including global trade Provide environmental footprint information of food products to consumers Transition to sustainable food systems within planetary boundaries
Biodiversity Strategy	SDG2: sustainable food production SDG6: freshwater ecosystems SDG14: marine ecosystems	Reduction of the environmental footprint of food production Need for sustainable supply chains and consumption patterns that do not exceed planetary boundaries Better management of nitrogen and phosphorus throughout the life cycle of products Methods to measure the environmental footprint of food products
Circular Economy Action Plan	SDG6: water reuse and efficiency SDG7: energy efficiency SDG8: resource decoupling SDG9: sustainable industrialization SDG12: sustainable production SDG13: climate action	Producers' responsibility along the entire life cycle of products Provide environmental footprint information of products to consumers Reduction of EU consumption and material footprint, toward resource consumption within planetary boundaries Improved environmental footprint of key value chains
Chemicals Strategy for Sustainability	SDG2: sustainable food production SDG3: reduced exposure to toxic chemicals SDG6: improved water quality SDG9: sustainable industrialization SDG12: environmentally sound management of chemicals SDG14, 15: toxicity‐free environments	Promotion of technologies ensuring overall positive environmental and climate performance, from a full life cycle perspective Substances of concern are relevant through the life cycle of materials and products Chemical risk assessment should consider the whole life cycle of substances, materials, and products Reduced environmental footprint of chemical production

The policy documents included several aspects for which LCA might represent an appropriate method to support EU policies. First, the consideration of the entire life cycle of products is highlighted in all the communications. For example, the new Circular Economy Action Plan addresses key value chains instead of focusing on individual products and highlights the entire life cycle along the different legislative proposals, for example, “The possibility to introduce requirements linked to environmental and social aspects along the value chain, from production through use to end of life” (p. 6). In addition, in the Chemicals Strategy for Sustainability, the presence of toxic chemicals is not limited to the product itself but “through the life cycle of materials and products” (p. 7), where, for example, production processes and end‐of‐life management practices are also considered. Second, the role of the entire value chain and global trade is also emphasized in the assessed communications. For example, the Farm to Fork Strategy states that “As the biggest global food importer and exporter, the EU food and drink industry also affects the environmental and social footprint of global trade” (p. 12). On the contrary, the Biodiversity Strategy aims to “avoid or minimise the placing of products associated with deforestation or forest degradation on the EU market, and to promote forest‐friendly imports and value chains” (p. 22). Policies aim to better address the embedded impacts in imports, with potential new policy instruments that consider the environmental impacts of imported raw materials and products, for example, environmental taxes also being under consideration. Recent studies assessing the environmental impacts of EU consumption outlined the relevance of embedded impacts in trade (Beylot et al., 2019; Corrado et al., 2020; Sanyé Mengual & Sala, 2021). Hence, EU consumption is responsible for environmental impacts in other world regions where the imported goods are produced. This was highlighted in the last EU SDG monitoring report, which included the first attempt to evaluate spill‐overs: the economic benefits generated by EU imports in other world regions lead to negative environmental and social impacts as trade‐offs (Eurostat, 2021). The European Green Deal (EC, 2020a) recognized the need for better addressing transboundary effects associated with trade and EU consumption patterns. The role of global trade in the environmental impacts of countries has been outlined in the literature, such as in material footprints (Wiedmann et al., 2015) or in the eutrophication impacts associated with phosphorous (Hamilton et al., 2018). Therefore, the consideration of the entire life cycle of products through methods such as LCA is essential for systematically including the different supply chains of products and services in their evaluation.

Third, many policy objectives aim to reduce the environmental impacts of the addressed systems (e.g., EU food system) and products (e.g., chemical production). For this purpose, a quantitative method is essential for allowing the calculation of baselines and assessing the progress over time. Defining baselines can support setting specific quantitative targets, as well as monitoring the efficacy of implemented policies. In this sense, LCA, as a systematic and quantitative method, can provide an assessment of the EF at the macro‐scale level to support different steps of the policy cycle. As a tool of the Better Regulation Toolbox (EC, 2021b), LCA can be used in support of EU policy development (e.g., definition of emerging problems, identification of policy options), including setting baselines to test different policy options.

Finally, the assessed policy documents enhance the use of EF as a basis for business‐to‐business or business‐to‐consumers information. The Commission already targeted using the EF method when launching the Single Market for Green Products Initiative (EC, 2013) and the related Product and Organisation Environmental Footprint (PEF/OEF) methods (EC, 2021c). PEF/OEF are LCA‐based methods that further prescribe a number of rules to be applied when assessing products and organizations aiming to set specific guidelines for comparative EFs among the EU market. Currently, there is a transition phase in which the methodology is further tested prior to the possible adoption of policies implementing the PEF/OEF methods. The EF is indeed mentioned explicitly in the Circular Economy Action Plan (“companies substantiate their environmental claims using Product and Organisation Environmental Footprint methods” [p. 6]).

### Assessing sustainable production and consumption (SDG12) with the LCA‐based Consumption Footprint and linkage with EU policy goals

The assessment of specific SDGs might be more comprehensive with the development of focused LCA‐based indicators addressing impacts along value chains. The Consumption Footprint indicator was developed with the goal of monitoring SDG12 at the macroscale (Sala, Benini, et al., 2019; Sala & Castellani, 2019). Therefore, the analysis of the performance of the indicator over time allows for monitoring the environmental impacts resulting from EU consumption patterns and intensity. A decrease in the indicator values would indicate a shift toward a more sustainable consumption in the EU. A proposal to use such an indicator is made in the monitoring of the new Circular Economy Action Plan, where the need to reduce the EU consumption footprint is specifically mentioned: “Updating the Circular Economy Monitoring Framework to reflect new policy priorities and develop further indicators on resource use, including consumption and material footprints” (annex to the Communication, p. 4). This communication aims to complement the material footprint indicator, which has been used as a pressure‐level indicator to track resource efficiency, with the goal of including an indicator covering not only resource use but also emissions to the environment at the impact level (i.e., reflecting the fact that environmental pressures of the same typology such as resource use have a different impact intensity).

On the basis of the Consumption Footprint indicator, the Consumer Footprint Calculator allows individual citizens to address SDG12 in relation to their own lifestyle (EC‐JRC, 2021). This tool can support the new Circular Economy Action Plan by enhancing the awareness of citizens of their individual consumption footprint and identifying the consumption patterns in which more sustainable actions are required, thereby contributing to the empowerment of consumers in the green transition.

**Figure 2:**
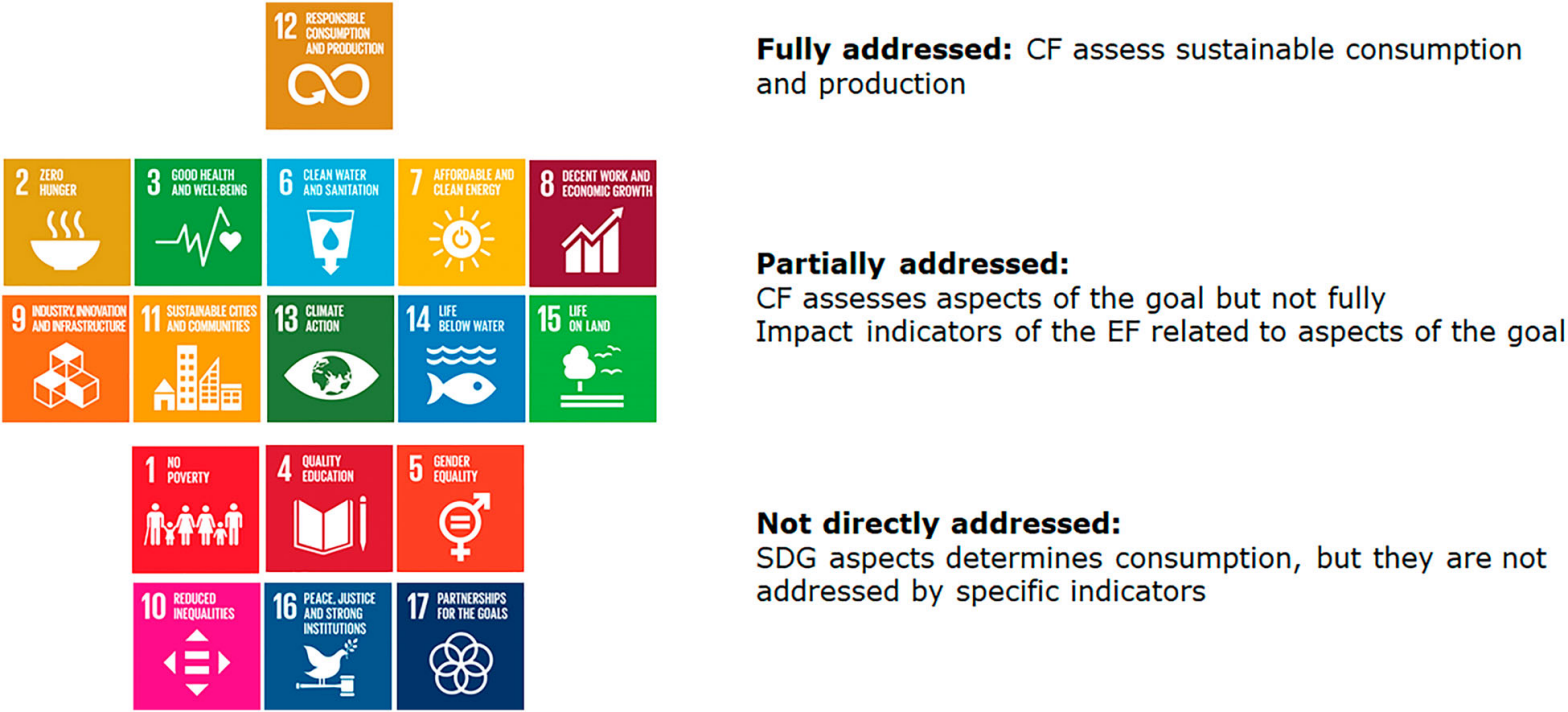
Coverage of Sustainable Development Goals (SDGs) by the Life Cycle Assessment (LCA)‐based Consumption Footprint (CF) indicator. The United Nations permits the use of SDG icons for informative use (data based on Sanyé‐Mengual et al., 2019a)

Beyond assessing SDG12 at the goal level, a detailed analysis on how the Consumption Footprint covers the SDGs framework from the goal to the indicator level (Sanyé‐Mengual et al., 2019a) revealed that it partially quantitatively addresses other eight SDGs at the goal level (Figure 2). While six SDGs are not directly addressed by the Consumption Footprint, these are associated with social and economic aspects determining the socioeconomic context influencing consumption patterns. In this sense, socio‐economic SDGs are indirectly addressed in lifestyles analyzed by the Consumption Footprint. The interlinkages between environmental and social indicators are qualitatively addressed in the above‐mentioned KnowSDGs platform.

**Figure 3:**
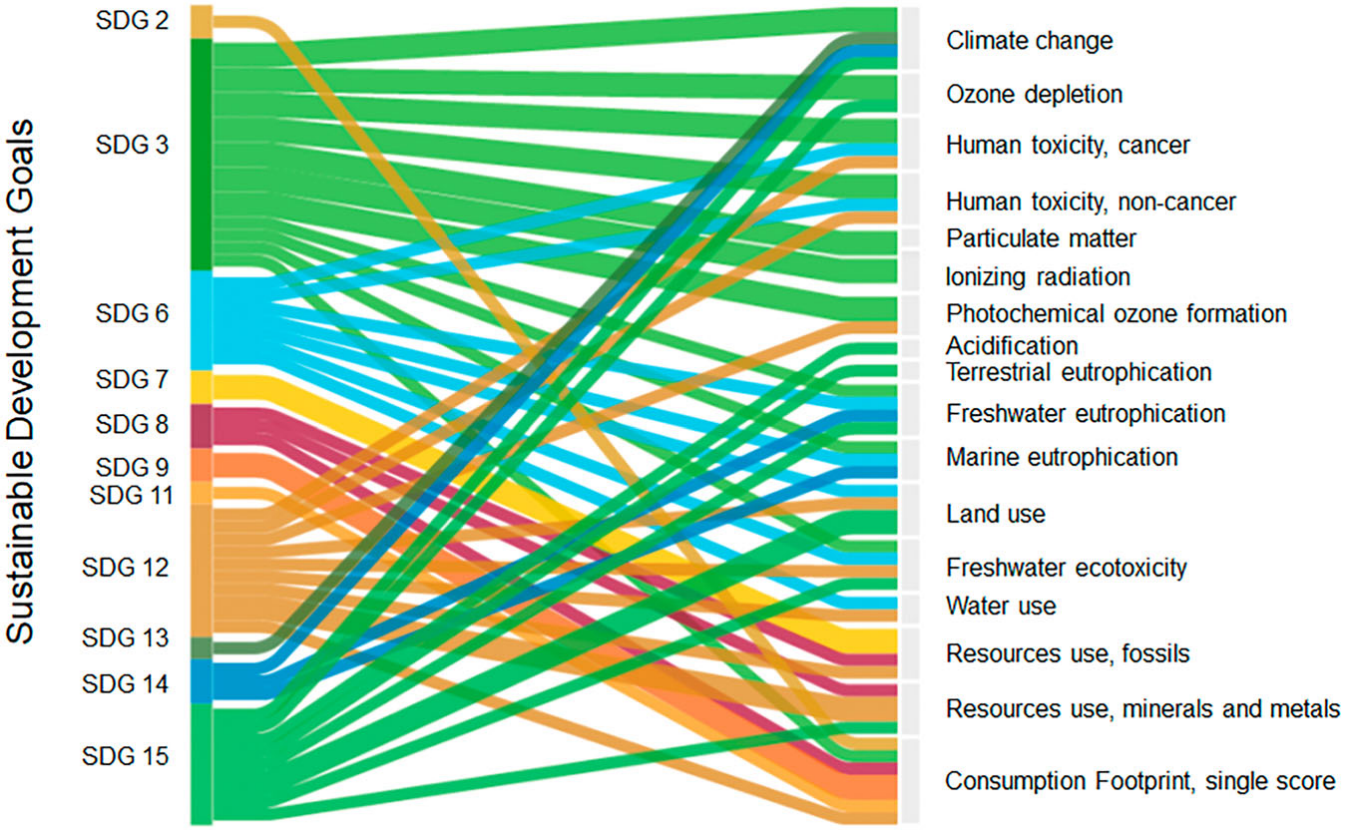
Relationship between Sustainable Development Goal (SDG) targets and impact indicators of the Environmental Footprint method (data based on Sanyé‐Mengual et al., 2019a)

When assessing the link between SDG targets and the EF impact indicators, it is revealed that although the Consumption Footprint was designed to address SDG12, the specific targets of SDG12 are only covered partially. However, the use of the EF as an LCIA method addressed targets from multiple SDGs (Figure 3). At the indicator level, the different impact categories of the EF method used in the Consumption Footprint were mapped with the SDG indicators. Although the Consumption Footprint was only linked to some SDG indicators, such as the ones related to resource efficiency, various environmental impact categories might be used as complementary indicators for some targets of SDG12. For example, the impact categories addressing toxicity impacts might be used to address target 12.4 on sustainable management of chemicals. This broader coverage of SDGs outlines the link with specific aspects addressed in the analyzed EU policies. Table 2 analyzes the specific links between the SDGs covered by the 16 impact categories of the Consumption Footprint and specific environmental issues addressed by the analyzed EU policies. This evaluation highlighted that the five SDGs are associated through the LCIA categories with environmental issues tackled by the selected communications, with CC and ECOTOX being common in the four documents.

**Table 2:** Link between Sustainable Development Goals (SDGs), Environmental Footprint impact categories, and environmental issues addressed in the analyzed EU (European Union) policies: new Circular Economy Action Plan (CEAP), Farm to Fork (F2F) Strategy, Chemical Strategy for Sustainability (CSS), and Biodiversity Strategy (BS)

SDG	Environmental Footprint impact category	CEAP	F2F	CSS	BS
3	Human toxicity (cancer)	x		x	
	Human toxicity (noncancer)	x		x	
	Particulate matter				
	Photochemical ozone formation				
	Ionizing radiation				
6	Water use	x	x		x
	Freshwater ecotoxicity	x	x	x	x
13	Climate change	x	x	x	x
	Resource use, fossil	x			
	Ozone depletion potential				
14	Marine eutrophication	x	x		x
	Freshwater eutrophication	x	x		x
15	Land use		x		x
	Terrestrial eutrophication	x	x		x
	Acidification				x
	Resource use, minerals, and metals				x

**Figure 4:**
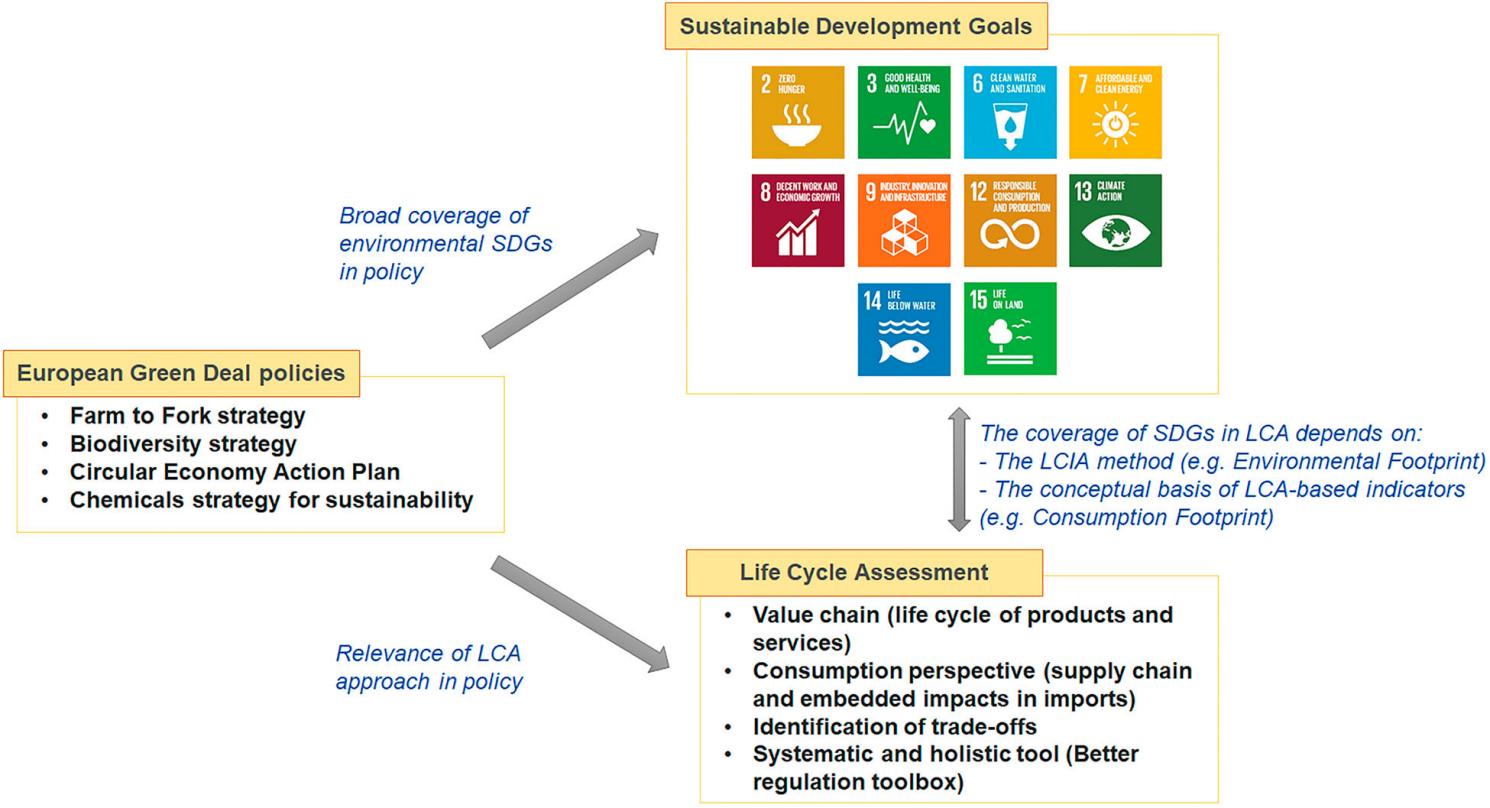
Interlinkages between European Green Deal policies, Sustainable Development Goals (SDGs), and Life Cycle Assessment (LCA). The United Nations permits the use of SDG icons for informative use

Therefore, there are close interlinkages between LCA, EU policies, and SDGs. However, the specific coverage of SDGs in LCA depends on the LCIA method used and the scope of the LCA study or LCA‐based indicator. In the case of the Consumption Footprint and the EF method, there is a broad coverage of SDGs (Figure 4). In fact, the use of the Consumption Footprint would enable the use of a life cycle and consumption‐based approach to assess SDG12, considering the relevance of such aspects in current EU policies. Furthermore, the Consumption Footprint indicator may help in overcoming the identified limitations of current SDG12 indicators in addressing the impacts of resource consumption, the different environmental impacts of each resource, and the lack of coverage of spill‐over effects, which has been highlighted in the literature (Schmidt‐Traub et al., 2017). One aspect to be considered when assessing SDGs using LCIA methods is the uncertainty and the potential effect that a more limited accuracy might have in the assessment of trade‐offs. In the case of the EF, impact categories are associated with a level of recommendation based on the robustness and uncertainty of the underpinning impact assessment methods (Fazio et al., 2018).

### Relative and absolute sustainability perspectives

The assessment of sustainability can be performed from both relative and absolute perspectives. Exploring the example of the Consumption Footprint, between 2010 and 2015, this metric increased between 3% (HTOX_c and WU) and 10% (RMD) (Table 3). This increase is associated with higher consumption of products, also resulting from the population increase (i.e., the accession of Croatia to the EU and the overall increase over time). The highest increase in resource use (minerals and metals) highlights the growing trend in the use of electronics (Sala, Benini, et al., 2019).

**Table 3:** EU Consumption Footprint results and gross domestic product (GDP) for 2010 and 2015, and change over time (%) (adapted from Sala, Benini, et al., 2019)

Impact category	Unit	2010	2015	Change (%)
CC	kg CO_2_ eq	4.80E+12	5.05E+12	5
ODP	kg CFC11 eq	3.22E+06	3.48E+06	8
HTOX_nc	CTUh	2.51E+05	2.65E+05	5
HTOX_c	CTUh	7.14E+04	7.35E+04	3
PM	Death	3.68E+05	3.88E+05	6
IR	kBq U^235^ eq	2.43E+11	2.53E+11	4
POF	kg NMVOC eq	1.30E+10	1.38E+10	6
AC	molc H^+^ eq	3.73E+10	3.99E+10	7
TEU	molc N eq	1.26E+11	1.37E+11	9
FEU	kg P eq	4.66E+08	5.10E+08	9
MEU	kg N eq	1.18E+10	1.28E+10	9
ECOTOX	CTUe	7.46E+12	8.04E+12	8
LU	kg soil loss	1.19E+13	1.27E+13	7
WU	m^3^ water eq	7.05E+12	7.29E+12	3
FRD	MJ	6.00E+13	6.24E+13	4
MRD	kg Sb eq	2.52E+07	2.78E+07	10
GDP	million €	1.28E+07	1.49E+07	16

Abbreviations: AC, acidification; CC, climate change; FEU, eutrophication, freshwater; HTOX_c, human toxicity, cancer; HTOX_nc, human toxicity, noncancer; IR, ionizing radiation, human health; MEU, eutrophication, marine; OdP, ozone depletion; PM, particulate matter; POF, photochemical ozone formation, human health; TEU, eutrophication, terrestrial.

While the evolution of the Consumption Footprint might be perceived as negative due to an increased intensity of environmental impacts in terms of time trends, an assessment of the decoupling between the environmental impacts and economic growth revealed a more positive message (Sanyé‐Mengual et al., 2019b). During the same time period, the EU's gross domestic product (GDP) increased by 16% and had a higher increase rate than any of the environmental impacts assessed. Therefore, when compared to the evolution of EU economic growth, the results indicate that environmental impacts of EU household consumption are decoupling relatively from economic growth as their rate of increase is lower than the GDP rate. A relative decoupling outlines the fact that environmental impacts are actually not decreasing but still increasing over time, although at a slower pace than economic growth. Ideally, environmental impacts should decrease, leading to an absolute decoupling. These results are in line with the relative and absolute decoupling of EU consumption in the last decades.

**Figure 5:**
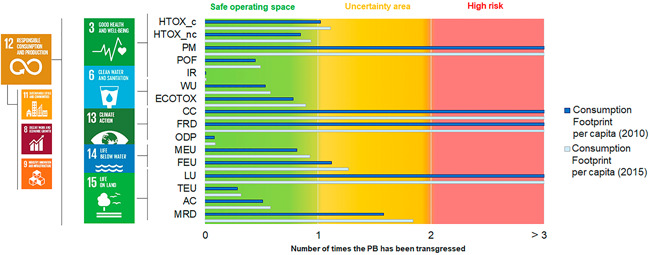
Consumption Footprint per capita (2010 and 2015) against the Life Cycle Assessment‐based Planetary Boundaries (PBs) per capita (data based on Sala, Benini, et al., 2019)

However, an absolute sustainability perspective can shed light on the current situation against the physical limits of the planet. Figure 5 shows the assessment of the Consumption Footprint per capita in 2010 and 2015 against the LCA‐based PBs. The environmental impacts of EU Consumption Footprint per capita transgressed the safe space for humanity of PM, CC, fossil resource use, and LU. The impacts of human toxicity (cancer), freshwater eutrophication, and mineral resource use are found in the uncertainty area, which provides a range of uncertainty to the estimated global planetary boundary. Therefore, although the EU Consumption Footprint showed a positive relative trend in terms of decoupling, the assessment against the PBs highlights the large contribution to specific environmental impacts of current consumption patterns that puts the resilience of ecosystems at risk. Moreover, an absolute sustainability assessment may identify environmental issues that should be better tackled in policy (e.g., CC in the high‐risk area), as the framework highlights that all nine PBs should be respected to guarantee correct functioning of the ecosystems (Rockström et al., 2009; Steffen et al., 2015).

The comparison of 2010 and 2015 in the assessment of the Consumption Footprint per capita against the PBs shows an increase from 2010 to 2015. This is not only related to an increased environmental impact (Table 2) but also the allocated global PBs (Rockström et al., 2009; Steffen et al., 2015) to the EU. In this study, this allocation has been performed per capita, thereby using an equality principle, where all global inhabitants have the same right, as widely implemented in similar studies (Häyhä et al., 2016). As well, it follows a static approach with 2010 as reference year and not reflecting changes along time. Due to a lower increase rate of the EU population compared to the global population, the share of PBs allocated to the EU region decreased between 2010 and 2015. Although different allocation principles might lead to variations of this output, an equality approach highlights the potential shrinkage of the limits of the planet allocated to the EU in the future.

The comparison of relative and absolute sustainability perspectives supports the ambition of EU policies to consider the planetary limits in environmental policy. Absolute sustainability can provide a reference to evaluate systems and define policy targets. The integration of the PBs framework into LCA can support the implementation of absolute sustainability in the context of EF and consumption footprint studies with a supply chain perspective. However, this might be limited by the LCIA method choice and the availability of an LCIA‐based PB set aligned with the metric, such as for the EF method.

When focusing on the integration of the PBs in EU policies and the SDGs framework, the evaluated EU policies reiterated the need to adopt transformational pathways that maintain the EU's footprint within the limits of the planet, thus considering that an absolute sustainability perspective in policymaking is crucial and, for example, the PBs framework can support the definition of science‐based quantitative targets (Sala et al., 2020). For example, the new CEAP cited the PBs: “To fulfill this ambition, the EU needs to accelerate the transition towards a regenerative growth model that gives back to the planet more than it takes, advance towards keeping its resource consumption within planetary boundaries” (p. 3). On the contrary, the current definition of the SDGs framework excludes the physical boundaries of the planet leading to the potential obstacle that identified efforts in the SDGs targets might not be enough to prevent environmental deterioration. For example, current SDG6 targets might not be met within the safe operating space for humanity (Sørup et al., 2020). Therefore, targets and indicators of the SDGs framework should be revisited toward better integrating the PBs.

## CONCLUSIONS

This study explored the potential role of LCA in supporting EU policies and achieving the SDGs through quantitative metrics as well as considering an absolute perspective through frameworks such as the PBs. To our knowledge, this is the first analysis in the literature addressing the interlinkages between LCA, EU policy, SDGs, and the PBs. This assessment is timely due to the higher relevance of LCT and LCA in recent EU policies within the European Green Deal.

**Figure 6:**
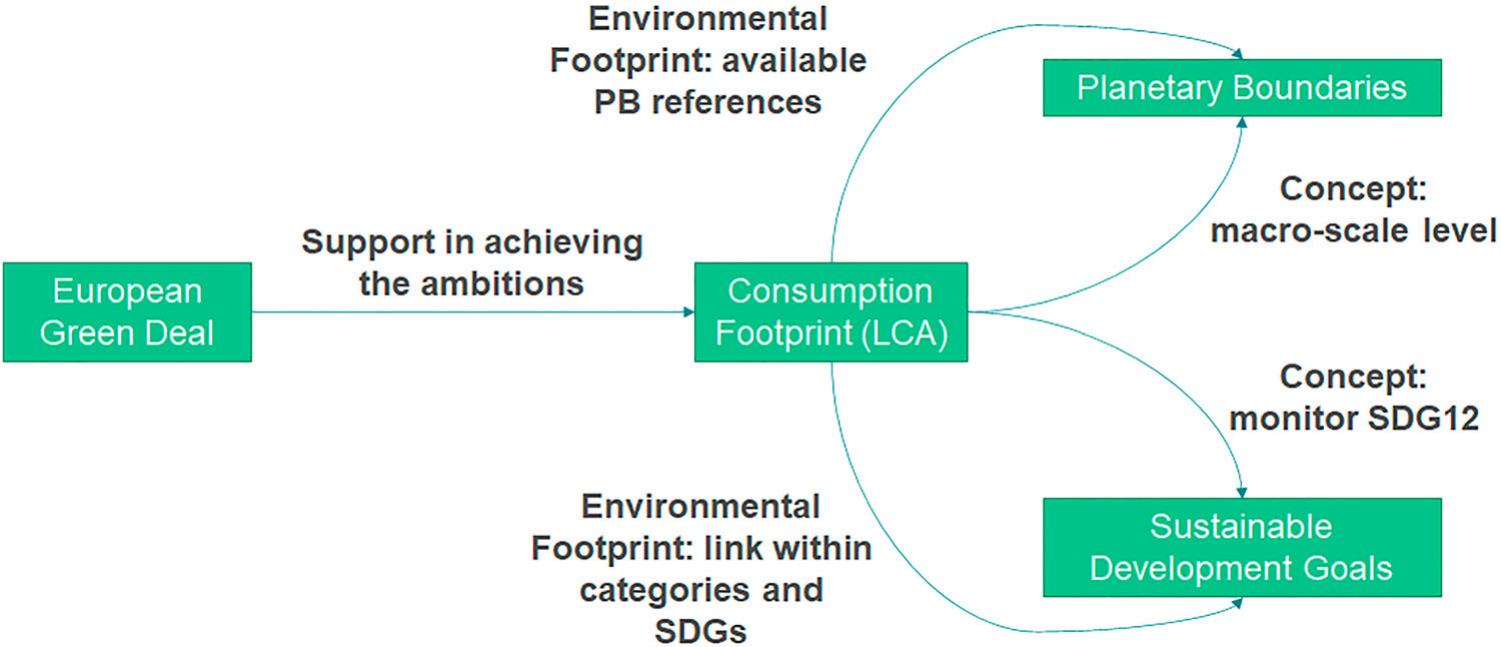
Life Cycle Assessment (LCA) can strengthen the linkages between EU (European Union) policy, Sustainable Development Goals (SDGs), and the Planetary Boundaries: the example of the Consumption Footprint

Relevant aspects associated with LCA have been highlighted in the analyzed EU policies: the relevance of supply and value chains, the necessity to consider traded goods in the assessment of footprints, the goal of reducing the EF of products and the EU consumption footprint, and the willingness to inform consumers about the EF of products. Furthermore, the analysis revealed that the four evaluated policies targeted specific elements of environment‐related SDGs, which can be covered by LCA and assessed against the PBs. However, the level to which an LCA framework can support EU policies in addressing SDGs and PBs depends on the scope of the study or indicator and on the LCIA method used (Figure 6). The example of the Consumption Footprint highlighted the role of the concept underpinning the LCA framework: this LCA‐based indicator, which was designed to quantitatively monitor SDG12, demonstrates that specific goals can be addressed by more comprehensive indicators. Furthermore, by using the EF LCIA method, it covers environmental aspects from CC (SDG13) to human toxicity (SDG3), LU (SDG15), or WU (SDG6), and it can be assessed against the PBs with the available set of EF‐based PBs. Therefore, LCA can support the contribution of EU policies to the SDGs while remaining within the PBs, although this is conditioned to the choice of the LCA methodology (e.g., conceptual basis of the framework, selected LCIA method).

The role of different sustainability perspectives was exemplified with the assessment of the EU Consumption Footprint. Relative sustainability can be used for analyzing time trends or the decoupling of environmental impacts from economic growth. However, absolute sustainability can provide a reference to evaluate systems and define targets. In fact, EU policy documents have reiterated the relevance to consider the limits of the planet in policymaking. In this context, the use of the PBs framework in LCA, as in the EF method, can operationalize an absolute sustainability perspective in quantitative analysis supporting policymaking. However, the analysis unveiled that further efforts are required in the SDGs framework to better integrate an absolute sustainability perspective, such as the provided by the PBs framework, toward aligning targets and indicators to the physical limits of ecosystems.

Life Cycle Assessment can be a pivotal method embedding a value chain and consumption approach that is currently highlighted in EU policies supporting SDGs and aimed at reducing environmental impacts to remain within the limits of our planet. The example of the Consumption Footprint highlights that the concept and LCIA methods of an LCA framework can determine the linkage among EU policy, SDGs, and the PBs. While this study focused on environmental sustainability, the assessment of social and economic SDGs might be performed in relation to social‐LCA and Life Cycle Costing methodologies, toward a broader coverage of the SDG framework. Furthermore, the use of the Consumption Footprint on specific geographic levels can support the focus on specific SDGs, such as applications at the city level to support SDG11 (Genta et al., 2022).

## CONFLICT OF INTEREST

The authors declare that there are no conflicts of interest.

## DISCLAIMER

The peer review for this article was managed by the Editorial Board without the involvement of S. Sala.

## Data Availability

Data are presented in the manuscript, with indicated references to underpinning studies (https://eplca.jrc.ec.europa.eu/sustainableConsumption.html).
